# *Portulaca oleracea* Polysaccharides Modulate Intestinal Microflora in Aged Rats *in vitro*

**DOI:** 10.3389/fmicb.2022.841397

**Published:** 2022-03-04

**Authors:** Qiang Fu, Siyi Zhou, Mengting Yu, Yang Lu, Genhe He, Xiaoliu Huang, Yushan Huang

**Affiliations:** ^1^College of Medicine, Jinggangshan University, Ji’an, China; ^2^Ji’an Key Laboratory of Biomedicine, Ji’an, China; ^3^School of Life Sciences, Jinggangshan University, Ji’an, China; ^4^Center for Evidence Based Medical and Clinical Research, First Affiliated Hospital of Gannan Medical University, Ganzhou, China

**Keywords:** *Portulaca oleracea* polysaccharides, *in vitro* fermentation, aged rats, intestinal microflora, probiotics

## Abstract

To explore the effect of *Portulaca oleracea* polysaccharides (POP) in regulating intestinal microflora in aged rats *in vitro*, its intestinal microbial composition was analyzed by 16 S rDNA high-throughput sequencing, and the level of short-chain fatty acids in fermentation broth was determined by LC-MS. POP significantly upregulated the relative abundance of *Lactobacillus*, *Eggerthella*, and *Paraprevotella* and significantly downregulated *Escherichia_Shigella*, *Bacteroides*, and *Eubacterium nodatum* groups. The pH value and ammonia nitrogen level decreased significantly in the POP-treated group, resulting in a more short-chain fatty acid consumption which changed the acid–base environment of the fermentation broth. In conclusion, POP is beneficial to aged rats because it can regulate intestinal flora, promote the growth of probiotics, and inhibit the reproduction of pathogenic bacteria.

## Introduction

The intestinal flora of mammals are colonized by a large number of microflora, whose homeostasis is of great significance to the physiological functions of the organism (Simpson and Campbell, 2015; Zhou et al., 2020). However, with aging, the intestinal flora of the organism also changes, with a decrease in the stability of the dominant species, especially in the elderly population of human beings, thus threatening their health (Salazar et al., 2020). Studies have shown that intestinal flora are closely linked to the development of aging-related diseases, which correlate with reduced intestinal probiotics (Zhou et al., 2020; Chu et al., 2021). In addition, several studies have provided scientific evidence that the abundance of *Bifidobacteria* decreased, whereas *Firmicutes* and *Enterobacteriaceae* increased in the intestines of elderly individuals compared with young individuals (O’Callaghan and van Sinderen, 2016). Studies have also found that the proportions of *Bacteroidetes*, *Firmicutes*, and *Verrucomicro* phyla in the intestinal tract diminished with age (Vemuri et al., 2017; Yu et al., 2017; Lee et al., 2018). The intestinal flora plays an important symbiotic function by producing short-chain fatty acids through fermenting low- or non-digestible carbohydrates that are not easily absorbed by the small intestine (Zhu et al., 2021).

Polysaccharides are complex carbohydrates made up of more than 10 monosaccharide molecules polymerized by glycosidic bonds. In recent years, abundant evidence has demonstrated that polysaccharides can be used as a carbon source of intestinal flora to promote the reproduction of probiotics and consequently regulate intestinal flora through its metabolites, which are vital in reshaping the diversity of intestinal microecology, making it a useful prebiotic (Fang et al., 2019). One of the resistant starches, amylose–lipid complex RS5, was reported to be helpful in regulating the microecology of human intestinal flora by promoting the production of butyric acid during *in vitro* fermentation, where the relative abundance of *Bifidobacterium*, *Dialister*, *Collinsella*, *Romboutsia*, and *Megamonas* increased significantly (Qin et al., 2021). In fact, acetic acid, propionic acid, and butyric acid regulate intestinal flora by reducing pH, inhibiting the excessive growth of pathogenic bacteria, such as *Escherichia coli*, and stimulating the growth of probiotics such as *Firmicutes* (Ghosh et al., 2011; Zimmer et al., 2012). Short-chain fatty acid production is considered a marker of interactions between host and intestinal flora, which also uncovers the causality between accelerated aging and flora dynamics in elderly individuals (Mangiola et al., 2018).

Purslane, a medicinal plant, is widely used in Traditional Chinese Medicine (Zhou et al., 2015). As a common homology of medicine and food, it also has a variety of indications (Iranshahy et al., 2017). Purslane is rich in bioactive substances, multiple flavonoids, alkaloids, polysaccharides, fatty acids, terpenoids, steroids, and proteins and is high in vitamins and minerals (Hu et al., 2018; Miao et al., 2019). Polysaccharides are the main active ingredients that have been widely studied. Results of the structural analysis show that glucose and galactose are the main monosaccharide molecules of *Portulaca oleracea* polysaccharide (POP). Research has demonstrated that POP exhibits antitumor and antimicrobial activities, improves diabetes, and promotes oxidation resistance (Dong et al., 2010; Hu et al., 2018). Our recently published study revealed that POP significantly improved the gut microbiota composition of weaned rats and promoted colonization probiotics such as *Lactobacilli* and *Bifidobacteria*, thus affecting the metabolic function of rats (Huang et al., 2021). This suggests that POP may play a role as a prebiotic in promoting the reproduction of intestinal probiotics and modulating the diversity of intestinal microecology. However, there are limited studies on the effect of POP as a prebiotic in gut microbiota homeostasis of aging individuals. The *in vitro* fermentation model is also considered to be an effective tool for investigating colon microflora in a highly controlled environment. Therefore, in the present study, based on our former results, we explored the effect of POP on regulating intestinal microflora of elderly rats *in vitro*, which provides potential insight into the development and utilization of POP as food.

## Materials and Methods

### Reagents

Purslane polysaccharides (≥50%) are purchased from Lanzhou Wotelaisi Biotechnology Co., Ltd. (Wotls, Lanzhou, China). Methanol, distilled deionized water, formic acid, and acetonitrile (HPLC grade) were purchased from Thermo Fisher Scientific (Waltham, MA, United States); 3-NPH, EDC, and standard products (HPLC grade) were purchased from Sigma-Aldrich (St. Louis, MO, United States). Related analytical reagents were purchased from Sinopharm Chemical Reagent Co., Ltd (Shanghai, China). The basic medium was prepared as the methodology reported by Mandalari et al. (2008) with minor modifications (2 g/l peptone, 2 g/l yeast extract, 0.1 g/l NaCl, 0.04 g/l K_2_HPO_4_, 0.04 g/l KH_2_PO_4_, 0.01 g/l MgSO_4_⋅7H_2_O, 0.01 g/l CaCl_2_⋅6H_2_O, 2 g/l NaHCO_3_, 2 ml Tween 80, 0.02 g/l hemin, 10 μl vitamin K_1_, 0.5 g/l cysteine HCl, and 0.5 g/l bile salts, pH 7.0).

### *In vitro* Fermentation of Purslane

Fecal samples collected by aseptic manipulation from five healthy Sprague-Dawley (SD) aged (21-month-old) rats were pooled with equal amounts and immediately placed inside an anaerobic chamber. 5 g of fecal samples was taken, and 20 times of the fecal weight of sterile saline was added to the anaerobic chamber, and the mixtures were fully shaken. The mixtures were filtered with eight layers of sterile gauze and transferred into another sterile sample bottle and then seeded into the basic medium to a final volume of 1 l for bacteria fermentation. The experiments were divided into six groups: Con1 (control group, fermented for 24 h), Con2 (control group, fermented for 48 h), LPOP1 (low-dose group of purslane polysaccharide fermented for 24 h), LPOP2 (low-dose group of purslane polysaccharide fermented for 48 h), HPOP1 (high-dose group of purslane polysaccharide fermented for 24 h), and HPOP2 (high-dose group of purslane polysaccharide fermented for 48 h). The fermentation volume of all groups was in 10 ml fermentation solution, the Con groups contained no POP, the LPOP groups contained POP (0.02 g/ml), and the HPOP groups contained POP (0.03 g/ml). Each group was repeated with five independent experiments. Gas production of the anaerobic cultures was recorded at 0, 12, 24, 36, and 48 h post inoculation (hpi), and the fermentation culture was collected at 24 and 48 hpi. Supernatants of the culture were separated by centrifugation for detection of pH, ammonia nitrogen, and short-chain fatty acid levels. The precipitates were stored at −80°C for further DNA extraction.

### Analysis of Ammonia

For ammonia analysis, 2.5 ml phenol chromogenic agent was added into a 10-ml tube, and 10 μl fermentation supernatants was added later. Then, 2.0 ml hypochlorite solution was also added into the tube. After shaking and mixing, the tube was incubated in water bath at 40°C for 15 min then cooled to room temperature to measure the absorbance value (OD_625_), and the ammonia content was calculated according to the standard curve.

### Determination of Short-Chain Fatty Acids

Short-chain fatty acids were analyzed by LC-MS/MS. A volume of 100 μl supernatant was added with 100 μl 50% acetonitrile-aqueous solution (v/v), and then grinding for 3 min. After that, the samples were sonicated in an ice water bath for 10 min and then centrifuged for 10 min, at 4°C, 12,000 rpm, followed by sample derivation and stored at −80°C for further analysis. The 5-μl samples were added into a high-performance liquid chromatograph (Nexera UHPLC LC-30A, Shimazin, Japan) with a color matching column (ACQUITY UPLC BEH C18 100 m × 2.1 mm × 1.7 μm, Waters) and a high-resolution mass spectrometer (AB SCIEX QTRAP 5500, AB SCIEX). The mass spectrum conditions are as follows: 0.1% formic acid-aqueous solution (A) and acetonitrile (B) are used as mobile phase, mass flow rate is 0.35 ml/min; curtain gas: 35 (psi); collision-activated dissociation (CAD) parameters: medium; negative ion spray voltage: −4,500 (V); ion source temperature: 450(°C); column temperature: 40(°C), ion source: gas 1:50 (psi); gas 2:60 (psi). Various short-chain fatty acids were determined according to the retention time, and the concentrations were calculated based on the standard curve.

### DNA Extraction and Gene Amplification

Cecal content samples of the rats were collected and quick-frozen, then stored at −80°C for further analysis. Total genomic DNA was extracted using the DNA Extraction Kit (Magen, Guangzhou, China) according to the manufacturer’s instructions. The concentration of DNA was verified with a NanoDrop 2000 spectrophotometer (Thermo Fisher Scientific, Waltham, MA, United States) and agarose gel electrophoresis, respectively. All DNA samples were stored at −20°C for further study. PCR amplification of the V3–V4 hypervariable regions of the bacterial 16S rRNA gene was carried out with a 25-μl reaction mixture using universal primers (343F: 5′-TACGGRAGGCAGCAG-3′; 798R: 5′-AGGGTATCTAATCCT-3′). The reverse primer contained a sample barcode, and both primers were connected with an Illumina sequencing adapter.

### Library Construction and Sequencing

The amplicon quality was visualized by gel electrophoresis. The PCR products were purified with Agencourt AMPure XP beads (Beckman Coulter Co., Brea, CA, United States) and quantified using the Qubit dsDNA assay kit. The concentrations were then adjusted for sequencing. Sequencing was performed on an Illumina NovaSeq 6000 with two paired-end read cycles of 250 bases each (Illumina Inc., San Diego, CA, United States; Oe Biotech Company, Shanghai, China).

### Bioinformatic Analysis

Paired-end reads were preprocessed with Trimmomatic software (Bolger et al., 2014) to detect and cut off ambiguous bases (N). It also cut off low-quality sequences with an average quality score below 20 using a sliding window trimming approach. After trimming, paired-end reads were assembled using FLASH software (Reyon et al., 2012). Parameters of assembly were 10 bp of minimal overlapping, 200 bp of maximum overlapping, and 20% of maximum mismatch rate. Further denoising of sequences was performed as follows: reads with ambiguous, homologous sequences or below 200 bp were abandoned. Reads with 75% of bases above Q20 were retained by QIIME software (version 1.8.0) (Caporaso et al., 2010). After that, reads with chimera were detected and removed using VSEARCH (Rognes et al., 2016). Clean reads were subjected to primer sequence removal and clustering to generate operational taxonomic units (OTUs) using VSEARCH software with a 97% similarity cutoff (Rognes et al., 2016). The representative read of each OTU was selected using the QIIME package. All representative reads were annotated and blasted against Silva database (Version 132) using the RDP classifier (confidence threshold was 70%) (Wang et al., 2007). The microbial diversity in cecal content samples was estimated using the alpha diversity that includes Chao1 index, Shannon index, Observed-species, Simpson, Goods-coverage, and PD-whole-tree. The UniFrac distance matrix performed by QIIME software was used for unweighted UniFrac principal coordinate analysis (PCoA) and phylogenetic tree construction. The 16S rRNA gene amplicon sequencing and analysis were conducted by Oe Biotech Co., Ltd. (Shanghai, China).

### Statistical Analysis

Experimental data in the study are reported as mean ± standard deviation (SD) of at least biological duplicates. One-way analysis of variance (ANOVA) and Duncan’s multiple-range tests were employed to analyze significant differences in gas production, NH_3_-N value, pH value, short-chain fatty acid (SCFA), and α-diversity (*p* < 0.05) between groups using SPSS version 19.0 (SPSS Inc., Chicago, IL, United States). Gas, NH_3_-N, pH, and SCFA profiles were plotted in the GraphPad Prism version 8.0 (GraphPad Software, Inc., La Jolla, CA, United States).

## Results

### Gas Production, NH_3_-N Value, and pH Value Analysis

**FIGURE 1:**
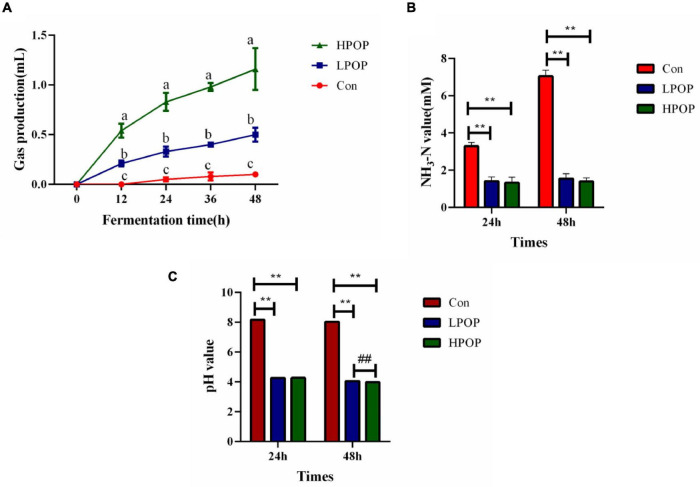
Gas production of the anaerobic cultures was recorded at 0, 12, 24, 36, and 48 h post inoculation (hpi), and the fermentation culture was collected at 24 and 48 hpi. Supernatants of the culture were separated by centrifugation for detection of pH and ammonia nitrogen levels. Gas production, NH_3_-N, and pH value during 48 h *in vitro* fecal fermentation with POP. **(A)** Gas production. **(B)** NH_3_-N. **P < 0.01 and ^##^*P* < 0.01 vs different groups, respectively. **(C)** pH value.

As fermentation time increased, gas production in the HPOP and LPOP groups changed significantly compared with that in the Con group. Moreover, gas production in the HPOP group was significantly higher than in the LPOP and Con groups (Figure 1A). At the 24- and 48-h fermentation points, both ammonia nitrogen (NH_3_-N) and pH values were significantly higher in the Con group than in the HPOP and LPOP groups (Figures 1B,C).

**FIGURE 2:**
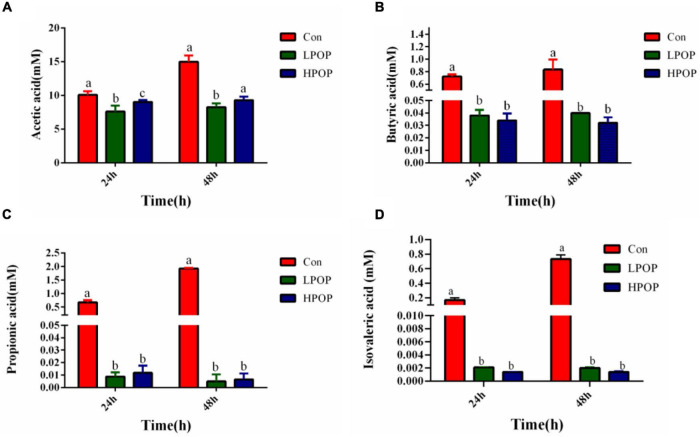
Acetic acid **(A)**, butyric acid **(B)**, propionic acid **(C)**, and isovaleric acid **(D)** production after 24 and 48 h *in vitro* fecal fermentation of POP.

The results also showed that POP inhibited the production of short-chain fatty acids in the fermentation broth. After 24 and 48 h of fermentation, the concentrations of acetic acid (Figure 2A), butyric acid (Figure 2B), propionic acid (Figure 2C), and isovalerate (Figure 2D) in the fermentation broth of POP groups (both low- and high-dose groups) significantly decreased compared with Con groups; however, there was no difference between the LPOP and HPOP groups (Figure 2).

### Effects of *Portulaca oleracea* Polysaccharides on Microbial Community Distribution

**FIGURE 3:**
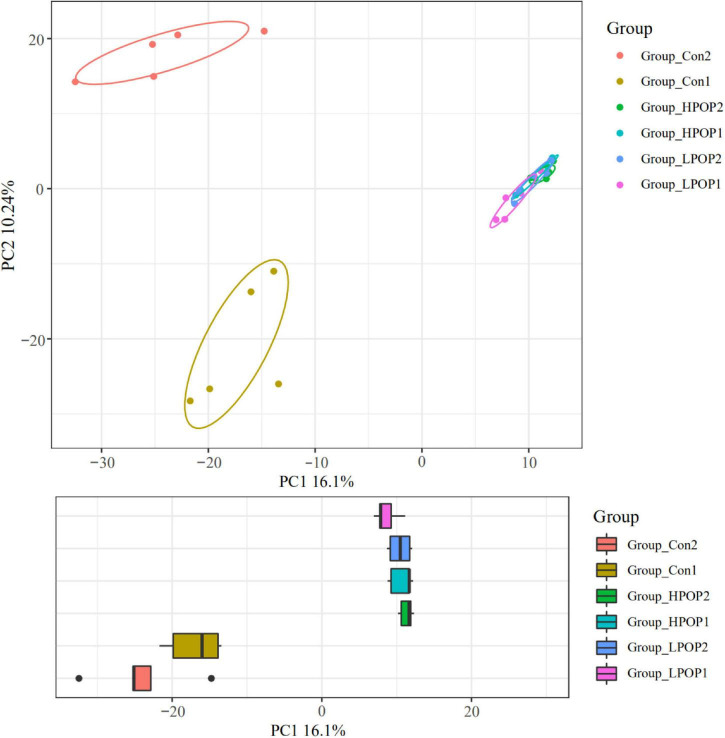
Principal component analysis (PCA) of the microbial community composition after 24 and 48 h *in vitro* fecal fermentation with POP.

The Good’s coverage indices in the six groups were all greater than 99%, which indicated that most of the bacteria present in the samples were identified (Table 1). Purslane polysaccharides were found to significantly decrease fecal microbiota richness (Shannon, Simpson, and PD-whole-tree) and diversity (Chao1 and observed-species) after 24 or 48 h of *in vitro* fermentation compared to the control groups (Table 1). In addition, POP altered bacterial β-diversity (Figure 3). Principal component analysis (PCA) revealed the distinct clustering of microbiota compositions for the six groups. The microbiota compositions of LPOP1, LPOP2, HPOP1, and HPOP2 were not comparable; however, Con1 and Con2 were significantly comparable, but Con groups differed greatly from POP groups in the PC1 analysis, which was 16.1% of the total variation (Figure 3).

**TABLE 1:** α-Diversity after 24 and 48 h *in vitro* fecal fermentation of POP.

Sample	Chao1	Observed-species	Shannon	Simpson	Goods-coverage	PD-whole-tree
Con1	762.89 ± 16.74^a^	614.36 ± 25.81^a^	3.21 ± 0.31^a^	0.668 ± 0.074^b^	0.9968 ± 0.0001^a^	24.23 ± 0.84^a^
LPOP1	576.20 ± 41.15^b^	428.04 ± 36.33^b^	2.68 ± 0.11^b^	0.679 ± 0.042^b^	0.9973 ± 0.0002^b^	18.62 ± 1.56^b^
HPOP1	538.11 ± 18.94^b^	370.18 ± 40.07^c^	3.06 ± 0.42^ab^	0.778 ± 0.070^a^	0.9975 ± 0.0001^b^	16.51 ± 1.54^c^
Con2	765.34 ± 45.08	620.08 ± 42.26	3.59 ± 0.12	0.711 ± 0.033	0.9971 ± 0.0001	23.97 ± 1.72
LPOP2	528.94 ± 64.93*	382.30 ± 48.16*	2.57 ± 0.11*	0.686 ± 0.012	0.9976 ± 0.0004*	16.82 ± 1.96*
HPOP2	529.33 ± 41.76*	371.58 ± 35.76*	2.64 ± 0.44*	0.705 ± 0.072	0.9976 ± 0.0002*	16.47 ± 1.55*

*Values are mean ± SD.*

Significant (p < 0.05) differences among 24 h are indicated with different letters, *p < 0.05 means v Con2.

### Microbial Composition at Phylum and Genus Levels

**FIGURE 4:**
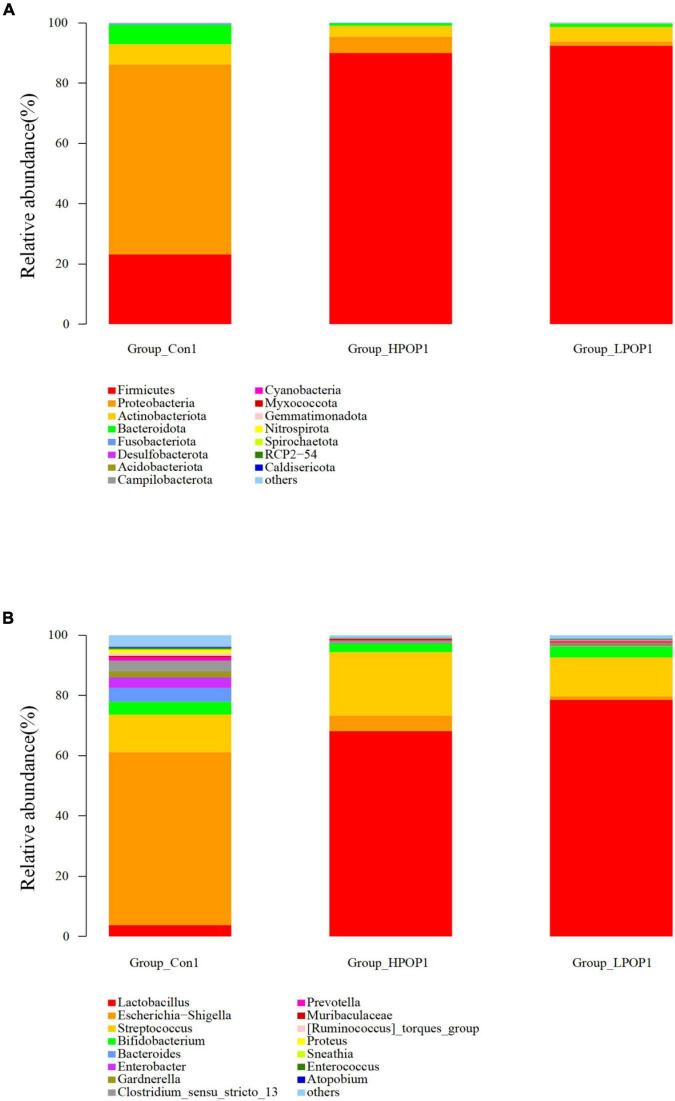
Effects of POP on microbial composition of the fermentation (phylum and genus levels). Changes of microbiota composition at phylum **(A)** and genus **(B)** levels at 24 h *in vitro* fecal fermentation with POP.

**FIGURE 5:**
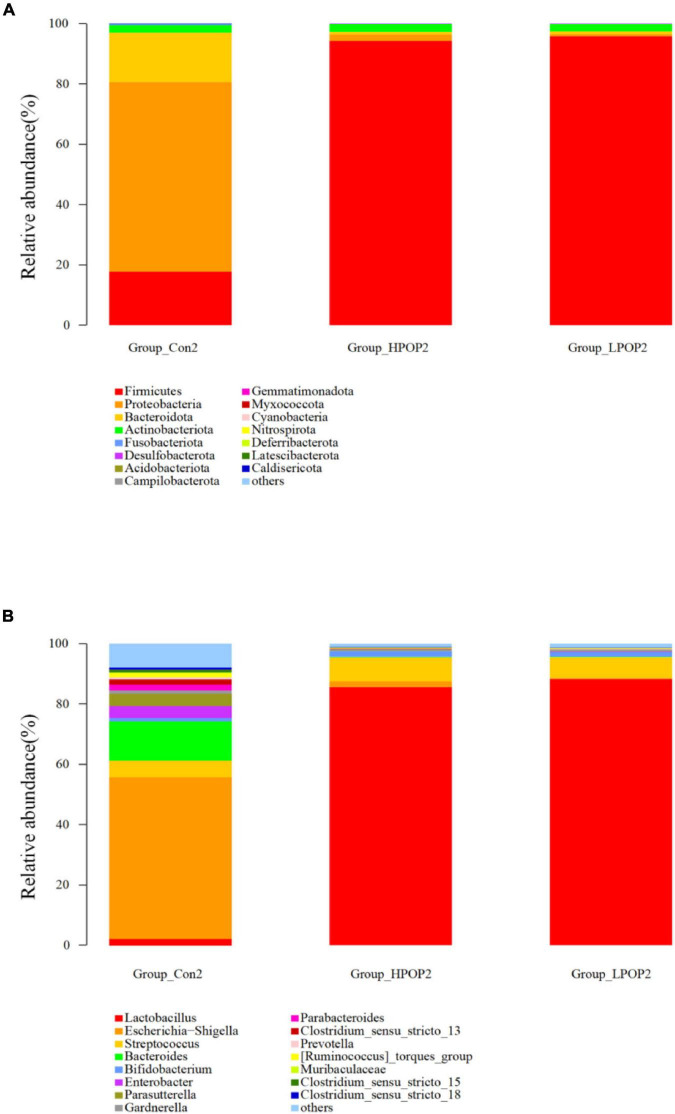
Effects of POP on microbial composition of the fermentation (phylum and genus levels). Changes of microbiota composition at phylum **(A)** and genus **(B)** levels at 48 h *in vitro* fecal fermentation with POP.

Microflora in fermentation cultures of different groups are mainly composed of *Firmicutes*, *Bacteroidetes*, *Proteobacteria*, and *Actinobacteriota* when categorized at the phylum level. Nine and six different phyla were observed with ANOVA analysis after 24 and 48 h of fermentation, respectively. Compared with the Con1 group, the abundance of *Firmicutes* in the LPOP1 and HPOP1 groups was significantly upregulated, while *Bacteroidetes*, *Proteobacteria*, *Fusobacteriota*, *Desulfobacterota*, *Actinobacteriota*, *Acidobacteriota*, *Cyanobacteria*, and others showed a significant decrease (Figure 4A). In addition, compared with the Con2 group, the relative abundance of *Firmicutes* in the LPOP2 and HPOP2 groups was significantly upregulated, whereas the relative abundance of *Proteobacteria*, *Desulfobacterota*, *Bacteroidota*, *Fusobacteriota*, and others was significantly decreased (Figure 5A).

At the genus level, *Escherichia-Shigella* and *Lactobacillus* were dominant in the Con and POP groups, respectively. After fermentation for 24 and 48 h, ANOVA analysis showed that 87 and 78 different bacterial genera were observed, respectively. Compared with the Con1 group, the relative abundance of *Lactobacillus* in the LPOP1 and HPOP1 groups increased significantly. However, other genera of microflora such as *Escherichia-Shigella*, *Parasutterella*, and *Klebsiella* decreased significantly (Figure 4B). Compared with the Con2 group, the relative abundance of *Lactobacillus* in the LPOP2 and HPOP2 groups was much higher. Conversely, the relative abundance of *Escherichia-Shigella*, *Parasutterella*, *Phascolarctobacterium*, *Eubacterium nodatum*_group, and *Klebsiella* decreased significantly (Figure 5B).

### Screening of Different Species Between Groups

**FIGURE 6:**
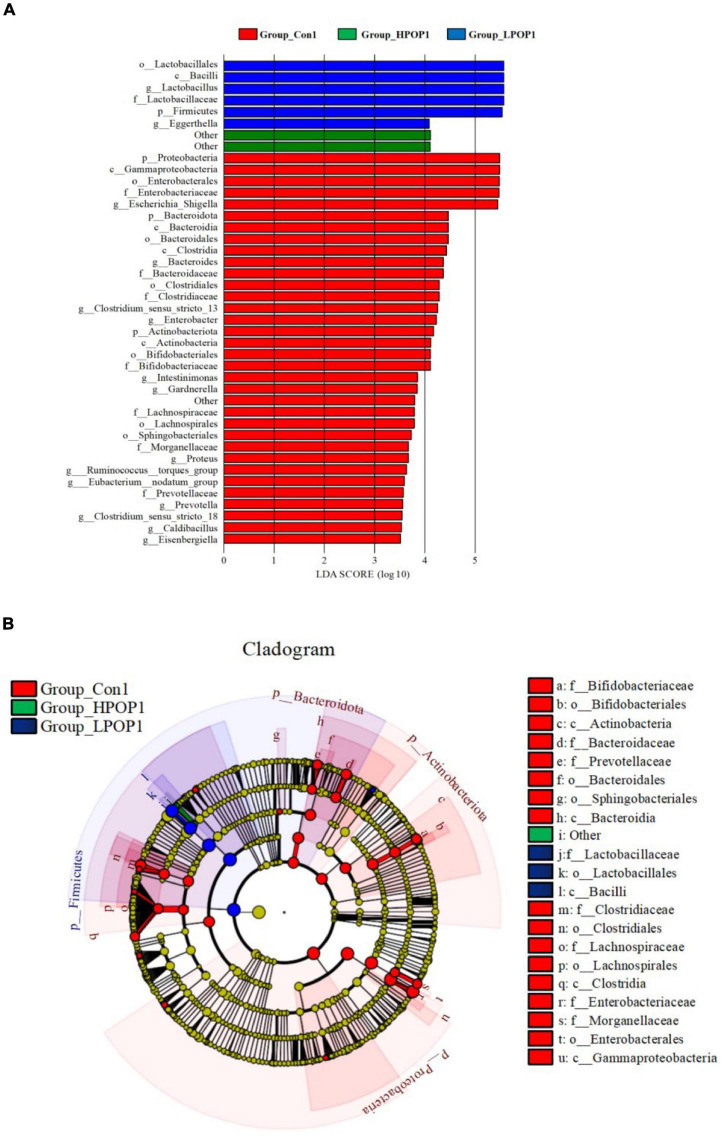
LEfSe analysis showing the most differentially abundant taxa (LDA score >3.0) after 24 h *in vitro* fecal fermentation with POP. **(A)** Histogram of the LDA scores. **(B)** Taxonomic cladogram of all differentially abundant taxa across all taxonomic levels [from the inner to outer rings, phylum (p), class (c), order (o), family (f), and genus (g)].

**FIGURE 7:**
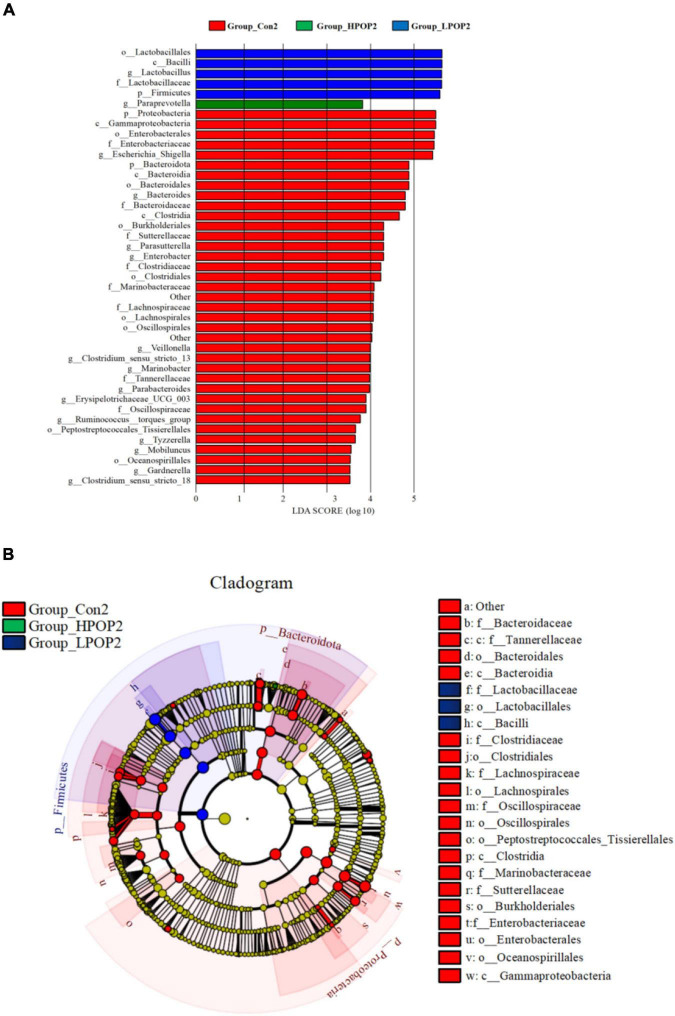
LEfSe analysis showing the most differentially abundant taxa (LDA score >3.0) after 48 h *in vitro* fecal fermentation with POP. **(A)** Histogram of the LDA scores. **(B)** Taxonomic cladogram of all differentially abundant taxa across all taxonomic levels [from the inner to outer rings, phylum (p), class (c), order (o), family (f), and genus (g)].

To identify specific microflora associated with POP, the linear discriminant analysis effect size (LEfSe) method was used to compare the differences in microflora between the Con and POP-treated groups after fermentation. The cladding diagrams of fermentative microorganisms and dominant bacteria composition are presented in Figures 6B, 7B. The most differentially abundant taxa are shown in Figures 6A, 7A. After 24 h of fermentation, the relative abundance of *Bacilli*–*Lactobacillus*, *Eggerthella*, and *Firmicutes* in the LPOP1 group was significantly increased, and in the HPOP1 group, the relative abundance of other bacteria was also significantly increased. In addition, the *Escherichia–Shigella*, *Bacteroides*, *Clostridium sensu stricto* 13, *Enterobacter*, *Intestinimonas*, *Gardnerella*, *Proteus*, *Ruminococcus torques group*, *Eubacterium_ nodatum group*, *Prevotella*, *Clostridium sensu stricto* 18, *Caldibacillus*, and *Eisenbergiella* in the Con1 group showed a significant increasing trend (Figure 6A). Furthermore, when the fermentation time was extended to 48 h, compared with the Con group, the relative abundance of *Bacilli–Lactobacillus* and *Firmicutes* in the LPOP2 group and *Paraprevotella* in the HPOP2 group showed a significant increasing trend. In the Con2 group, the relative abundance of several other phyla (i.e., *Escherichia–Shigella*, *Bacteroides*, *Parasutterella*, *Enterobacter*, *Veillonella*, *Clostridium sensu stricto* 13, *Marinobacter*, *Parabacteroides*, *Erysipelotrichaceae UCG-003*, *Ruminococcus_torques_group*, *Tyzzerella*, *Mobiluncus*, *Gardnerella*, and *Clostridium sensu stricto* 18) also significantly increased (Figure 7A).

### Functional Properties Inherent to the Microbiomes Using Phylogenetic Investigation of Communities by Reconstruction of Unobserved States

**FIGURE 8:**
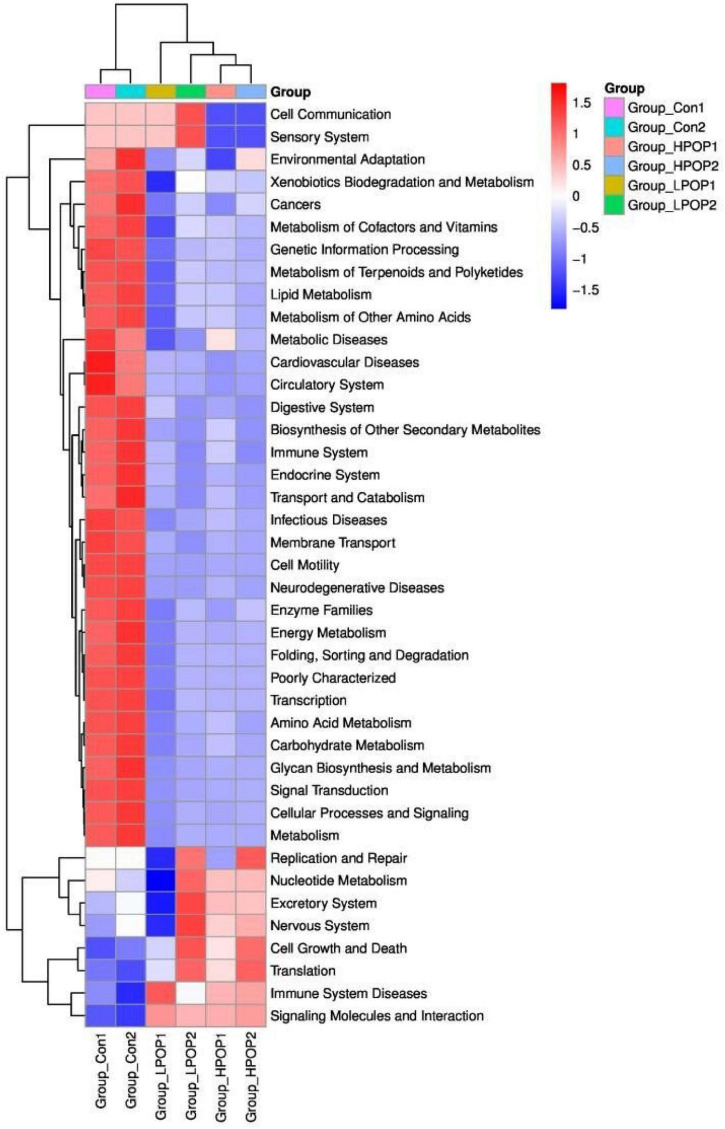
The heatmap showing the PICRUSt-predicted KEGG ortholog (KO) functions among the Con1, Con2, LPOP1, LPOP2, HPOP1, and HPOP2 groups in *in vitro* fermentation.

Phylogenetic Investigation of Communities by Reconstruction of Unobserved States (PICRUSt) is designed to predict metagenome functional content from marker gene surveys and full genomes. The marker gene of 16s rRNA was used to amplify the subsequence to estimate the metabolomic function spectrum of a microbial community. According to the PICRUSt results, there were significant differences in metabolic potential between the Con and POP groups (Figure 8).

Compared with the POP-treated group, the Con groups showed an increasing trend in the expression of genes associated with environmental adaptation, xenobiotics biodegradation and metabolism, metabolism of cofactors and vitamins, metabolism of terpenoids and polyketides, lipid metabolism, metabolism of amino acids, carbohydrate metabolism, glycan biosynthesis and metabolism, energy metabolism, folding, sorting and degradation, transport and catabolism, cellular processes, signaling and metabolism, membrane transport, cell motility, biosynthesis of other secondary metabolites, cancers, metabolic diseases, cardiovascular diseases, neurodegenerative diseases, infectious diseases, circulatory system, digestive system, immune system, endocrine system, enzyme families, poorly characterized transcription, signal transduction, and genetic information processing. Conversely, the expression of genes involved in cell growth and death, translation, immune system diseases, signaling molecules, and interaction decreased.

Regarding fermentation time, in both LPOP1 and LPOP2 groups, genes of replication and repair, nucleotide metabolism, excretory system, nervous system, cell growth and death, translation, immune system diseases, xenobiotics biodegradation and metabolism, metabolism of cofactors, cell communication, and sensory system showed a noticeable change in expression level. The expression of genes for environmental adaptation, replication, and repair in the HPOP1 and HPOP2 groups was also significantly changed.

## Discussion

The gut microbiome plays a crucial role in human health (Gomaa, 2020). There is increasing evidence that natural products and their bioactive compounds have aroused interest in their diverse bioactivities (Kim and Kim, 2018; Xu et al., 2018). Polysaccharides are functional natural products that have been widely studied. Studies have shown that polysaccharides that are hard to digest can improve sugar metabolism and prevent diseases by altering the composition of the gut microbiome (Wang H. et al., 2018; Wang M. et al., 2018). Our results demonstrate that the pH values of fermentation cultures in the LPOP and HPOP groups are significantly lower than those in the Con group at 24 and 48 h of fermentation. The alteration in the pH value is an indicator of the rate of the fermentation reaction. Thus, POP can accelerate the speed of fermentation, which is consistent with changes in gas production in the fermentation system. At each time point, the gas production in the POP groups is significantly higher than that of the Con groups. Moreover, with the increase in polysaccharide concentration, gas production also significantly increased, which indicates that POP is a good carbon source. NH_3_-N is a harmful metabolite generated by intestinal flora. Excessive NH_3_-N concentration leads to an increased ammonia metabolic load or ammonia poisoning (Luo et al., 2013). We found that NH_3_-N levels reduced significantly in POP-treated groups, which indicates that POP has a positive role in NH_3_-N emissions.

Furthermore, the α diversity and species richness of fecal flora decreased with POP fermentation at both low and high doses. Low α diversity may be associated with several processes, including efficient metabolism of POP, and/or increase in the relative abundance of the intestinal flora subgroup by POP degradation products, or suppression of the growth of flora that is sensitive to a low-pH environment (Duncan et al., 2009; Bendiks et al., 2020). With the decrease in overall microbial gene richness, the level of short-chain fatty acids in the POP groups decreased significantly. Compared with the Con groups, the yields of acetic acid, butyric acid, propionic acid, and isovaleric acid in POP groups were also significantly reduced at 24 and 48 h of fermentation time. A recent study found that the yield of SCFAs increased significantly in the first 12 h of resistant starch fermentation then decreased in the following 12 h (Qin et al., 2021). Similar results were also observed in the *in vitro* fermentation of other forms of dietary fiber (Carlson et al., 2017). Thus, we infer that the increase in fermentation time and the rapid proliferation of bacteria in the POP group result in nutrient and short-chain fatty acid consumption.

Consistent with the structure of the main flora in the human intestinal tract, the four phyla of *Bacteroidetes*, *Firmicutes*, *Proteobacteria*, and *Actinobacteria* are the most important in the intestinal tract of elderly rats in this study (Simpson and Campbell, 2015; Binda et al., 2018). *Firmicutes* and *Bacteroidetes* account for more than 90% of the relative abundance of intestinal flora, and interactions between these two phyla are vital in maintaining intestinal homeostasis (Simpson and Campbell, 2015; Binda et al., 2018). Actinomycetes and Proteobacteria account for the remaining 10% (Arumugam et al., 2011; Segata et al., 2012). *Firmicutes* are a phylum of gut microbiota and are composed of many different gram-positive bacteria. It is the main producer of butyrate in the gut and degrades indigestible polysaccharides. *Bacteroidetes* are mainly composed of gram-negative bacteria which are important in carbohydrate metabolism (Graf et al., 2015). *Proteobacteria* are composed of a variety of pathogenic bacteria, such as *Escherichia coli*, *Salmonella*, *Vibrio cholerae*, and *Helicobacter pylori*, which indicates a microecological disorder and is a potential diagnostic indicator of intestinal diseases (Shin et al., 2015; Rizzatti et al., 2017). Actinobacteria are mainly composed of gram-positive bacteria, including three major families of anaerobic bacteria (*Bifidobacteria*, *Propionibacteria*, and *Corynebacteria*) and one family of aerobic bacteria (*Streptomyces*) (Binda et al., 2018). Our present study demonstrates that POP treatment significantly promoted the relative abundance of *Firmicutes* while reducing the relative abundance of *Bacteroidetes*, *Actinobacteria*, and *Proteobacteria*, among which *Proteobacteria* showed the most significant decrease. These data suggest that POP can regulate the composition and function of intestinal flora by affecting the abundance of Firmicutes, which is consistent with the findings on the effects of POP on intestinal flora in weaned rats in our previous study.

To identify the specific microflora associated with POP effects, LEfSe analysis with a standard LDA score >3.0 was used to compare the microflora composition after 24 and 48 h of fermentation, to determine the group characteristics of microorganisms in each fermentative substrate. In contrast, the abundance of *Lactobacillus* dominated in the POP groups, while *Escherichia–Shigella* accounted for a high proportion in the Con groups. *Lactobacillus* belongs to the *Firmicutes* phylum, *Bacilli* class, and *Lactobacillus* family, acting as an essential regulator of homeostasis in the intestinal tract (O’Callaghan and O’Toole, 2013). It has an important role in reducing cholesterol, inhibiting the proliferation of harmful intestinal bacteria, promoting immunity, and facilitating the absorption of minerals and energy conversion (Shinohara et al., 2010; Pessione, 2012). *Paraprevotella*, a gram-negative anaerobe, was found to be significantly reduced in primary hypothyroidism patients (Su et al., 2020). Other studies have reported a high abundance of *Paraprevotella* and *Eggerthella* in patients with depression and an increased abundance of pro-inflammatory *Escherichia–Shigella* in patients with cognitive impairment and brain amyloidosis (Barandouzi et al., 2020). Likewise, our results show that POP can promote probiotic Lactobacillus in the intestinal flora of elderly rats and inhibit the reproduction of pro-inflammatory *Escherichia–Shigella*, which suggest that the intake of POP may be beneficial to the health of aging individuals.

The results from the PICRUSt analysis also reveal that after 48 h of fermentation, the proportion of genes associated with replication and repair, cell growth and death, translation, nucleotide metabolism, and excretory and nervous systems is relatively high. There is probably a connection with the increased relative abundance of *Firmicutes*, which metabolize starch, galactose, and butyric acid, especially with low-dose POP. However, the expression of genes associated with infectious diseases, metabolic diseases, cardiovascular diseases, cancers, and neurodegenerative diseases in the Con groups was significantly higher than that in the POP groups, which is consistent with the results that *Escherichia–Shigella* was the dominant flora in the Con groups.

## Conclusion

*Firmicutes*, *Bacteroidetes*, *Proteobacteria*, and *Actinobacteriota* are the main phyla of gut microbiota in elderly rats. However, the diversity and composition of rat gut microbiota are significantly altered after POP treatment. The intake of POP reduces the pH and NH_3_-N levels of fermentation substrates and consumes more short-chain fatty acids, which in turn promotes probiotics such as Lactobacillus and inhibits colonization of pathogenic bacteria such as *Escherichia–Shigella*.

This study provides new insights into the potential use of POP as a bioactive ingredient to maintain intestinal microbiota in a balanced state. Further studies should be conducted to investigate the potential physiological effects of POP fermentation on colon microecological health.

## Data Availability Statement

The datasets presented in this study can be found in online repositories. The names of the repository/repositories and accession number(s) can be found below: https://www.ncbi.nlm.nih.gov/, PRJNA789821.

## Ethics Statement

The animal study was reviewed and approved by the Committee on the Ethics of Animal Experiments of the Jinggangshan University.

## Author Contributions

QF and XH conceived, designed the experiment, and drafted the manuscript. SZ, MY, and YL performed the experiments and collected the data. GH, XH, and YH provided resources and reviewed the manuscript. All authors contributed to the article and approved the submitted version.

## Conflict of Interest

The authors declare that the research was conducted in the absence of any commercial or financial relationships that could be construed as a potential conflict of interest.

## Publisher’s Note

All claims expressed in this article are solely those of the authors and do not necessarily represent those of their affiliated organizations, or those of the publisher, the editors and the reviewers. Any product that may be evaluated in this article, or claim that may be made by its manufacturer, is not guaranteed or endorsed by the publisher.
